# Sympatric otariids increase trophic segregation in response to warming ocean conditions in Peruvian Humboldt Current System

**DOI:** 10.1371/journal.pone.0272348

**Published:** 2022-08-11

**Authors:** Susana Cárdenas-Alayza, Michael J. Adkesson, Mickie R. Edwards, Amy C. Hirons, Dimitri Gutiérrez, Yann Tremblay, Valentina Franco-Trecu

**Affiliations:** 1 Centro para la Sostenibilidad Ambiental, Universidad Peruana Cayetano Heredia, Lima, Peru; 2 Laboratorio de Ciencias del Mar, Universidad Peruana Cayetano Heredia, Lima, Peru; 3 UMR 248 MARBEC: IRD–Univ. Montpellier–CNRS–Ifremer, Sète cedex, France; 4 Chicago Zoological Society, Brookfield Zoo, Brookfield, Illinois, United States of America; 5 Halmos College of Arts and Sciences, Nova Southeastern University, Dania Beach, Florida, United States of America; 6 Programa Maestría en Ciencias del Mar, Universidad Peruana Cayetano Heredia, Lima, Peru; 7 Departamento de Ecología y Evolución, Facultad de Ciencias, Universidad de la República, Montevideo, Uruguay; MARE – Marine and Environmental Sciences Centre, PORTUGAL

## Abstract

Determining trophic habits of predator communities is essential to measure interspecific interactions and response to environmental fluctuations. South American fur seals, *Arctocephalus australis* (SAFS) and sea lions *Otaria byronia* (SASL), coexist along the coasts of Peru. Recently, ocean warming events (2014–2017) that can decrease and impoverish prey biomass have occurred in the Peruvian Humboldt Current System. In this context, our aim was to assess the effect of warming events on long-term inter- and intra-specific niche segregation. We collected whisker from SAFS (55 females and 21 males) and SASL (14 females and 22 males) in Punta San Juan, Peru. We used δ^13^C and δ^15^N values serially archived in otariid whiskers to construct a monthly time series for 2005–2019. From the same period we used sea level anomaly records to determine shifts in the predominant oceanographic conditions using a change point analysis. Ellipse areas (SIBER) estimated niche width of species-sex groups and their overlap. We detected a shift in the environmental conditions marking two distinct periods (P1: January 2005—October 2013; P2: November 2013—December 2019). Reduction in δ^15^N in all groups during P2 suggests impoverished baseline values with bottom-up effects, a shift towards consuming lower trophic level prey, or both. Reduced overlap between all groups in P2 lends support of a more redundant assemblage during the colder P1 to a more trophically segregated assemblage during warmer P2. SASL females show the largest variation in response to the warming scenario (P2), reducing both ellipse area and δ^15^N mean values. Plasticity to adapt to changing environments and feeding on a more available food source without fishing pressure can be more advantageous for female SASL, albeit temporary trophic bottom-up effects. This helps explain larger population size of SASL in Peru, in contrast to the smaller and declining SAFS population.

## Introduction

Individual foraging behavior and trophic interactions determine the flow of energy along the food web, and ultimately these drive population dynamics, community structure, and most ecosystem processes [1]. However, unpredictable and highly variable environments can also affect trophic behavior [2]. Top predators, such as pinniped species, are considered structural components of communities and ecosystem functioning [3]. Determining trophic habits of predators in a community is essential to measure the potential interspecific niche overlap and strength of interactions as well as the effects of environmental changes [4].

Two otariid species breed in sympatry along the coast of South America: the South American fur seal, *Arctocephalus australis* (SAFS), and the South American sea lion, *Otaria byronia* (SASL). Information from areas where these otariids coexist serves as an interesting model to assess interspecific segregation [5–7]. However, the majority of research has focused on the Atlantic Ocean where SAFS are recognized as pelagic and SASL as benthic; little is known about their segregation in the upwelling Humboldt Current System in the Pacific Ocean. In contrast to the Atlantic coast that has a wide continental shelf, Peru’s coast is characterized by a narrow continental shelf, productive upwelling waters, and relatively shallow thermocline and oxycline that are related with the upper boundary of the oxygen minimum layer at depths of approximately 50–80 m on average [8–11]. These features limit the potential habitat for otariid prey to be linked to the benthos [12,13], making pelagic resources commonly available for tertiary consumers of the Humboldt Current System [14,15]. Thus, in this productive, pelagic ecosystem foraging on highly patchy, dense prey resources near the surface may be more beneficial energetically, than searching for more evenly distributed, but less dense, prey resources on the benthos [16].

The Humboldt Current System is very dynamic since it is exposed to recurring El Niño Southern Oscillation (ENSO). ENSO is a coupled oceanic-atmospheric cycle of alternating warm El Niño and cold La Niña events that vary in intensity and duration and at the same time, is the most prominent climate signal on Earth [17]. Along the Eastern and Western Pacific, there are several ‘Niño regions’ of varying sizes and locations, where a system of oceanographic buoys monitor the intensity and duration of ENSO [18]. When the Humboldt Current System is affected by an El Niño event, sea level pressure drops, sea surface temperature rises, thermocline and oxycline deepen, and reductions of primary productivity occur in the marine environment, altering distribution of habitats and associated prey biomass. ‘Extraordinary’ El Niño events that occurred 1982–83 and 1997–98 are well documented for dramatically altering species composition, reducing zooplankton and fish biomass, and causing mortality in higher trophic levels [19–21]. However, the effects on trophic adaptation during weaker events that do not cause direct mortality, but impact reproduction, juvenile survival, and weaning have not been thoroughly investigated.

Global warming is currently altering marine ecosystems’ structure and driving species distributions to higher latitudes, thereby leading to altered interspecific interactions with unspecified consequences [22,23]. Climate driven shifts are more pronounced at the poles where deviations from long-term climatic variables are more striking [24,25]. Short term warming events, such as El Niño, give insights on the impacts and responses of marine ecosystems in the Eastern Pacific [26]. In the past decade a series of warming events have impacted the Southeastern Pacific that may have affected the organization of predator communities in the Peruvian Humboldt Current System.

Long-term warming climatic/oceanic events, or heatwaves known as “The Blob”, have impacted the Pacific Ocean in recent years [27]. The Blob was first detected in the Gulf of Alaska in 2013–2014 and is a result of a persistent pattern of higher than normal values of sea level pressure and weak coastal winds. In Peru, El Niño conditions occurred between 2014 and 2015, and were followed by the strong El Niño event of 2015–2016 that affected the entire Pacific Ocean [28]. This event was then followed by a ‘Coastal El Niño’ event that was only detected in the Niño 1+2 region (0–10° S, 90° W—80° W), during austral summer and early fall of 2017 [29,30]. All the aforementioned events involve the rise of sea level forced by the passage of remotely generated and coastal trapped waves in response to heat expansion at some stage of the event [31]. The thermocline and nutricline deepened, decreasing nutrient enrichment in the euphotic zone. During the 2017 Coastal El Niño, weak winds caused the reduction in vertical nutrient fluxes to the depleted euphotic zone [30]. The poorer surface nutrient concentrations cause a decrease of surface chlorophyll-a and phytoplankton biomass. In turn the oceanographic changes trigger a shift in community structure toward smaller phytoplankton which can have bottom-up effects on food and energy availability at higher trophic levels [26,32].

Upper trophic level predators, like pinnipeds, act as sentinels to trophodynamic and species assemblage changes that occur lower in the food web [33]. Analysis of the ratios of stable isotopes in different tissues has emerged as a key method to investigate the differences in the foraging ecology of pinnipeds [34]. The δ^15^N value is used to estimate the trophic position [35] and δ^13^C values predict the carbon sources used by the predators [36,37] at different temporal scales, depending on the moment of production and the turnover rate of the tissue analyzed [38]. The analysis of ratios of stable isotopes in metabolically inert tissues, such as tooth dentine and whisker keratin with continuous growth, represents sequential archives that allow inference of foraging strategies at the individual level over long periods of time [5,39]. Based on previous studies that estimated whisker growth rates in these otariids [40], each whisker gives information for up to 4.8 years [41]. In our study, long-term niche differentiation in otariids species and sex groups is investigated by examining the stable isotope ratios serially archived in whiskers of male and female South American fur seals and South American sea lions. We aim to understand how trophic niche segregation between species and sex groups is affected by body size and foraging tactics as reflected through the isotopic niches detected in whisker segments in relation to changing environmental conditions.

Several authors have reported relationships showing that larger animals tend to consume higher trophic level prey [42,43]. South American sea lions are considerably larger in body size than fur seals and both species have a strong sexual dimorphism, with males being much larger than females [44]; thus we expect that SASL males have the highest values of δ^15^N and that SAFS males have higher values than SAFS females. In terms of δ^13^C, we expect groups to follow the inshore-offshore δ^13^C gradient already reported in the northern Humboldt Current System [45]. Since otariid females perform feeding trips during lactation, which usually last ~11 months [46], we suspect they are restricted to foraging grounds close to the breeding colony compared to males [47,48]. Thus, we expect both otariid females to show more enriched δ^13^C values, indicative of higher productivity since they remain constrained closer to the land-based colony. In contrast, we expect males to have a wider range of δ^13^C from foraging more “freely” between coastal and pelagic zones.

As reported, the Peruvian coast has been affected by a decrease in the biomass of local prey and an impoverishment in lower trophic levels during 2014–2017 warming ocean events [49]. On a long-term timescale, we expect to find a reduction in the trophic position of otariids and a higher overlap in isotopic niche space during warmer events when pelagic (cold-water) resources are less available. Finally, due to the beforementioned energetic constraints linked to maternal attendance, we expect females will reflect a narrow range of isotopic signatures compared to males and therefore be less flexible to adapt to changes in the environment.

## Materials and methods

### Study site and sample collection

Whiskers were collected from lactating females and adult males chosen at random during pinniped health assessment campaigns between 2010 and 2019 at Punta San Juan (PSJ), a marine protected area located along the southern coast of Peru (15° 22’ S, 75° 11’ W). Whiskers were collected from 76 SAFS (55 females and 21 males) and 36 SASL (14 females and 22 males). Individuals were divided into four sex-species study groups (SAFS females, SASL females, SAFS males, SASL males).

Anesthesia of individual SASL and male SAFS was induced using a combination of midazolam, butorphanol, and medetomidine administered via plastic dart as previously reported [49,50]. Female SAFS were captured using a hoop net and then anesthetized with isoflurane gas (1–5% to effect) mixed with oxygen as previously reported [51]. Anesthesia was performed by a board-certified specialist in zoological medicine. Body weights were obtained using a tripod and field scale to the nearest 100 grams (Mini Crane Scale 300 kg, OCS-L). Whiskers, including the follicle, were removed manually. All animals were determined to be in good health by a veterinarian based on physical examination findings and veterinary assessment of routine blood parameters. Sampling and methodology were approved by the Peruvian government under research permits Resolución Jefatural No. 009-2010-, No.023-2011-, No. 022-2012-, No. 09–2013, No. 024–2014, No. 008-2015-, and 019-2016-SERNANP-RNSIIPG issued by the Peruvian National Service of Natural Protected Areas (Spanish acronym SERNANP) and the Peruvian Ministry of the Environment (Spanish acronym MINAM). Procedures were approved by the Ethics Committee of Universidad Peruana Cayetano Heredia (#005-02-18, #012-01-21). Importation of samples to the United States for analysis was authorized under Marine Mammal Protection Act permits 15471 and 19669.

### Sample analysis

Each whisker was scrubbed with an abrasive plastic pad and rinsed with deionized water to remove surface contaminants. Whiskers were thoroughly dried at 60° C for a minimum of 24 hours, measured and then cut into 2.5 mm fragments from base (proximal) to tip (distal), obtaining between 10 and 62 fragments, according to the total length of the whisker. Each fragment was coded from tip to base and related to the corresponding length of the pertaining whisker segment in a database. Every other fragment was prepared for stable isotope analysis, while the remaining fragments were reserved. We then subsampled each portion to obtain a mass of 0.6–0.8 mg (high precision microbalance Mettler Toledo MX5, precision = 1μg). Fragments were placed in individual tin capsules, pelletized, and then sent for stable isotope ratio analysis.

Samples were combusted and analyzed for δ^13^C and δ^15^N at the Smithsonian Institution’s Museum Conservation Institute (Suitland, MD) using a Thermo Delta V Advantage mass spectrometer in continuous flow mode coupled to a Costech 4010 Elemental Analyzer (EA) via a Thermo Conflo IV (CF-IRMS) to determine natural carbon and nitrogen abundance and their isotopic ratios with an analytical precision of ± 0.2 ‰ for both isotopes. A set of standards were run for every 10–12 samples. The standards included USGS40 and USGS41 (L-glutamic acid) as well as Costech acetanilide. All samples and standards were run with the same parameters; this included an expected reproducibility of the standards < 0.2 ‰ (1σ) for both δ^13^C and δ^15^N. Stable isotope values were expressed in terms of δ and were reported relative to the standard reference material, Vienna Pee Dee Belemnite (VPDB) standard for δ^13^C and atmospheric air (N_2_) for δ^15^N. The resulting isotope ratios for each whisker segment were converted and reported to the conventional values delta (δ) with the standard parts per thousand notation (‰): δX = (R_sam_ / R_st_) - 1, where X stands for ^13^C or ^15^N, R for ^13^C/^12^C or ^15^N/^14^N, R_sam_ for isotope ratio of the sample, and R_st_ for isotope ratio of the standard.

### Data analysis

#### Reconstructing time series of δ^13^C and δ^15^N

In whiskers, the most recent growth is located at the base of the whisker, and an individual whisker can represent several years’ growth [52–54]. Otariid whisker growth rate estimated from live wild South American fur seals of 0.08 mm/day [40] was globally applied to this study. Therefore, each 2.5 mm segment represents approximately 30 days and each whisker represents between 4–6 years of serial information. This growth rate is applied to estimate a date for each segment to a month and year, based on the sampling date. All the dated fragments of each individual seal are then reorganized into a time series of δ^13^C and δ^15^N. By analyzing the time series of individual seals’ stable isotope ratios, a monthly mean for δ^13^C and δ^15^N was registered per individual (i.e. month, year). Across the 2,941 fragments from 112 whisker samples, a total of 14 years of monthly stable isotope ratios were recorded. The time series for the whisker fragments was consolidated from January 2005 up to December 2019.

#### Environment: Sea level anomaly and sea surface temperature

Monthly records for sea level anomaly (SLA) for the latitude of our study area (15° South) and period for the reconstructed time series (January 2005 to December 2019), were extracted from the Copernicus ocean products repository (https://marine.copernicus.eu/). Monthly sea surface temperature (SST) records were collected by the naval base at San Juan de Marcona bay (Dirección de Hidrografía y Navegación, DHN), located less than 5 km from the sampling site during the study period. We constructed a monthly time series of SST during the fifteen-year period. SLA and SST were correlated (r = 0.74, p< = .001), which means that nearly 50% of the variance of SST was explained by the correlation. While SLA reflects heat expansion in the water column as modulated remotely by equatorial dynamics and is a proxy of thermocline depth at intra-seasonal to interannual time-scales [55], SST rather reflects seasonal insolation changes and coastal upwelling dynamics in the area. Since SLA is closely linked with the large-scale ENSO influence in the region, and SST is more subjected to short-term local upwelling variability, we selected SLA in order to characterize the oceanographic regime shifts at the regional level during the study period. We then conducted a change point analysis, to identify when the probability distribution of a stochastic process or time series changes, assuming that the variance is constant. Change point analysis was applied to the SLA monthly time series to distinguish one change in environmental conditions in the 2005–2019 time series using cpt.mean function [56] in R package changepoint [57].

#### Stable isotope ratios

First, we used Linear Mixed-Effects Models (LMM) [58] to examine the effect of body mass on the δ^13^C and δ^15^N values from each sex group, using individual ID as random effect. Since body mass at the time of whisker collection is affected by feeding habits during recent months, in the LMM we included values from the last three fragments analyzed from each individual, integrating feeding information from the last six months. Second, we used LMM to examine the differences of the average δ^13^C and δ^15^N values between groups (species and sex), periods, and their interactions as fixed effects. Models included individual identity as a random effect to account for repeated measures of each response variable on the different fragments of each whisker. We used a continuous autocorrelation function [58] to model the serial correlation of the set of values of the response variables at the individual level. We compared the global model (all fixed effects and their interactions) with models without interaction with the Akaike Information Criterion (AIC) using the ΔAIC > 2 criterion [59]. Selected models were subject to the customary residual analyses (results not shown) and were found to have a satisfactory fit.

#### Isotopic niche

Stable Isotope Bayesian Ellipses in R (SIBER) package [60] was used to estimate isotopic niche width at 75% (standard ellipses) for SAFS and SASL applying a Bayesian approach. Mean isotopic values for each individual were used to estimate the ellipses since whisker fragments are temporally correlated and do not comply with sample independence. This method is a Bayesian version of Layman metrics [4] that can incorporate uncertainties such as sampling biases and small sample sizes into niche metrics [60]. Based on Markov-Chain Monte Carlo simulation, the SIBER approach obtains measures of uncertainty to construct parameters of ellipses in a way similar to the bootstrap. Standard ellipse areas corrected for small sample size (SEA_C_) were used to compare the different sex-species groups (SAFS females, SASL females, SAFS males, SASL males) in the isotopic space and estimated the area of overlap of their isotopic niche using Maximum Likelihood standard ellipse areas (SEA_C_). Values estimated for the overlap of each group is expressed in percentage.

Then, Layman metrics were estimated for the otariid community (the four species-sex groups) by period. Layman metrics are δ^13^C range, δ^15^N range, total area of convex hull (TA), mean distance to centroid from means (CD), mean nearest neighbor distance of the means, (MNND), and the standard deviation of the nearest neighbor distance (SDNND) following Layman et al. (2007).

## Results

### Influence of body mass on stable isotope ratios

LMM from females of both species indicate that there is a positive correlation between body mass and δ^15^N values, but only at intraspecific level (p-value < 0.001 in both cases). While SASL females are twice the size of SAFS, the former had 2.22 ‰ lower in δ^15^N values (p-value < 0.001). In the male LMM, no body mass effect on δ^15^N was detected for either of the two species (p-values > 0.05 in both cases).

### Study periods

**Fig 1:**
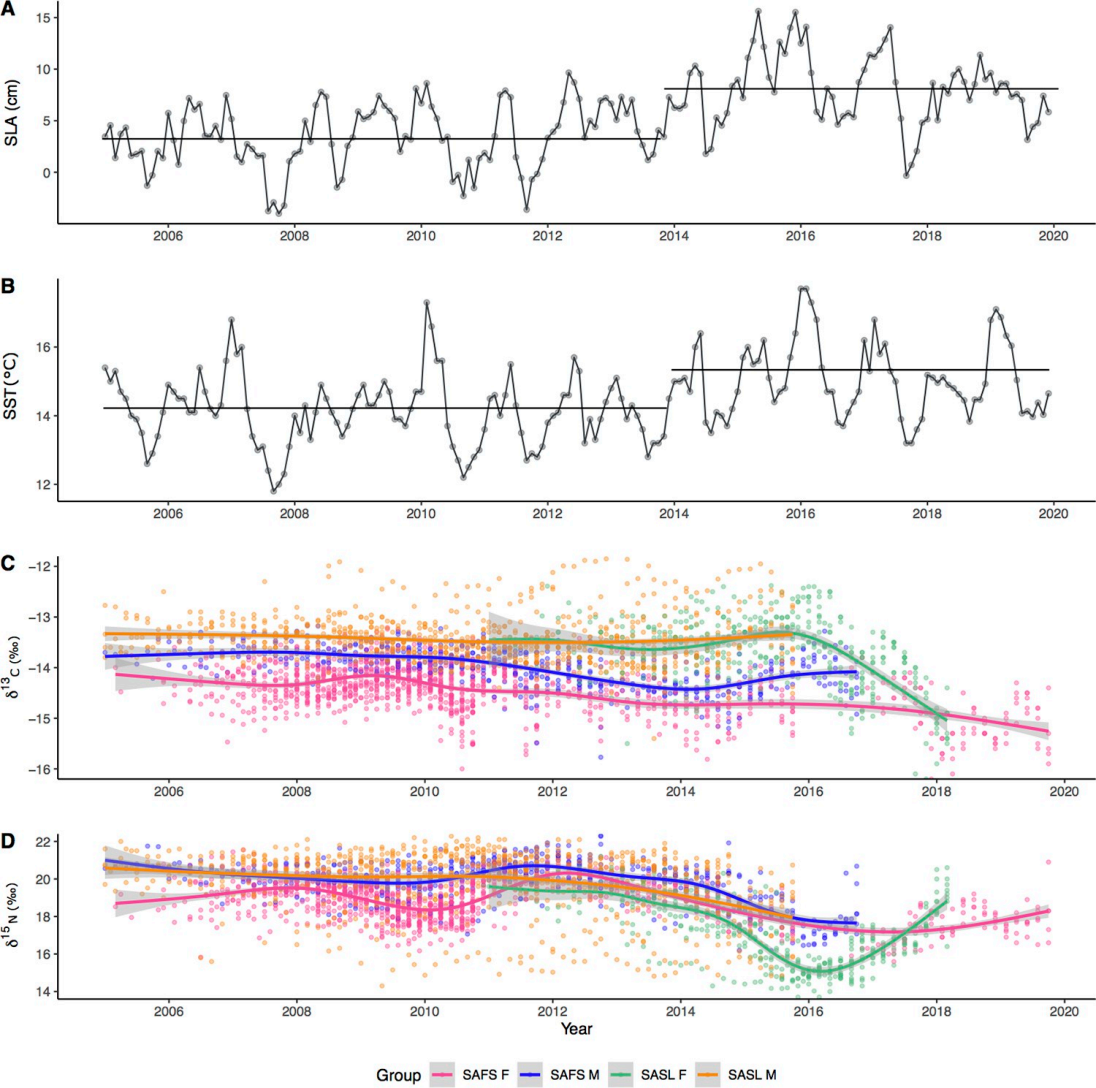
Time series for A) monthly sea level anomaly (SLA, cm) and D) sea surface temperature (SST,°C) from study area. Horizontal lines show start / end of Period 1 (mean = +3.4 cm) and Period 2 (mean = +8.1 cm) in SLA and start / end of Period 1 (mean = +14.1°C) and Period 2 (mean = +15.1°C) in SST. C) δ^13^C and D) δ^15^N isotopic signatures for all otariid groups.

We found a significant change point (p<0.05, MBIC = 15.58) in the SLA time series to divide it into two periods: Period 1, from January 2005 –October 2013 with a mean of +3.4 cm, and Period 2, from November 2013 –December 2019 with a mean of +8.1 cm (Fig 1A and 1B). A similar change point was found for SST (Period 1, mean of +14.1 cm and Period 2 mean of +15.1 cm) in SST, with only 1 month of difference (S1 Table), giving support to period breakdown. Therefore, in following analyses we compare changes between Period 1 and Period 2.

### Influence of environment on stable isotope ratios

The average lengths of the whiskers analyzed were 123.97 ± 11.72 mm and 175.63 ± 325.23 mm for SAFS and SASL, respectively. Mean isotopic values by group, sample size, and fragments number analyzed are shown in Table 1. There were significant differences in the mean whisker δ^13^C value among species-sex groups in both periods analyzed (see interaction terms in Table 2). In Period 1 the average δ^13^C was significantly lower for SAFS females compared with SAFS males and SASL males and females. However, the difference with SAFS males and SASL males is even greater in Period 2 due to the negative trend of the mean δ^13^C isotopic value of SAFS females in comparison to these groups (Fig 1, Table 2). Mean δ^15^N values significantly differed between species-sex group and periods, but without interaction. SASL females was the species-sex group that exhibited the largest decrease of the δ^15^N mean value in Period 2, and also showed a significant fluctuation (Fig 1, Table 2).

**Table 1:** Isotopic signatures of Peruvian otariid whiskers according to periods investigated in present study.

Species	Sex	*N*	Length (mm)	Period 1			Period 2		
				δ^13^C	δ^15^N	*N* fragments	δ^13^C	δ^15^N	*N* fragments
SAFS	F	55	115.68 ± 26.78	-14.32 ± 0.39	19.03 ± 0.94	1,002	-14.87 ± 0.47	17.99 ± 1.01	182
SASL	F	14	150.71 ± 32.61	-13.58 ± 0.50	19.05 ± 1.13	71	-13.72 ± 0.73	16.66 ± 1.60	322
SAFS	M	21	132.26 ± 40.34	-13.91 ± 0.35	20.20 ± 0.83	406	-14.27 ± 0.36	18.71 ± 1.30	125
SASL	M	22	200.54 ± 60.23	-13.45 ± 0.43	20.04 ± 1.54	719	-13.36 ± 0.58	18.58 ± 1.84	114
		112				2,198			743

Number of whiskers (*N*), mean length (mm) by species and sex. Mean ± SD of the stable isotope values (δ^13^C and δ^15^N) during two periods according to sea level. *N* fragments: Sample size for species and sex.

**Table 2:** Linear Mixed-Effects Models (LMM) for δ^13^C and δ^15^N values.

Model	Intercept	SAFS M	SASL F	SASL M	Period 2	SAFS M *Period2	SASL F *Period2	SASL M *Period2	AIC
**δ** ^**13**^ C~SpecieSex*Period	-14.40 (***)	0.35 (***)	0.47 (***)	0.88 (***)	-0.34 (***)	**0.17 (0.14)**	0.40 (***)	0.39 (***)	**1300**
δ^13^C~SpecieSex+Period	-14.44 (***)	0.37 (***)	0.69 (***)	0.96 (***)	-0.11 (***)	-	-	-	1311
δ^15^N~SpecieSex*Period	18.91 (***)	1.14 (***)	-1.02 (***)	0.86 (***)	-0.56 (0.0034)	-0.0008 (0.99)	-0.03 (0.92)	0.34 (0.21)	6291
**δ** ^**15**^ **N~SpecieSex+Period**	18.89 (***)	1.13 (***)	-1.10 (***)	0.94 (***)	-047 (***)	**-**	**-**	**-**	**6287**

LMM for δ^13^C_cor_ and δ^15^N_cor_ whisker values including group (sex-species), period and their interactions as fixed effects and identity and whisker portion as random effect (continuous autocorrelation function). Estimates and *p-values* (in brackets) are shown for each variable. In bold we show the selected model by the Akaike Information Criterion (AIC). Reference group for each predictor variable is SAFS F.

(***) denotes p<0.01.

### Isotopic niche size, overlap and community metrics according to period

**Fig 2:**
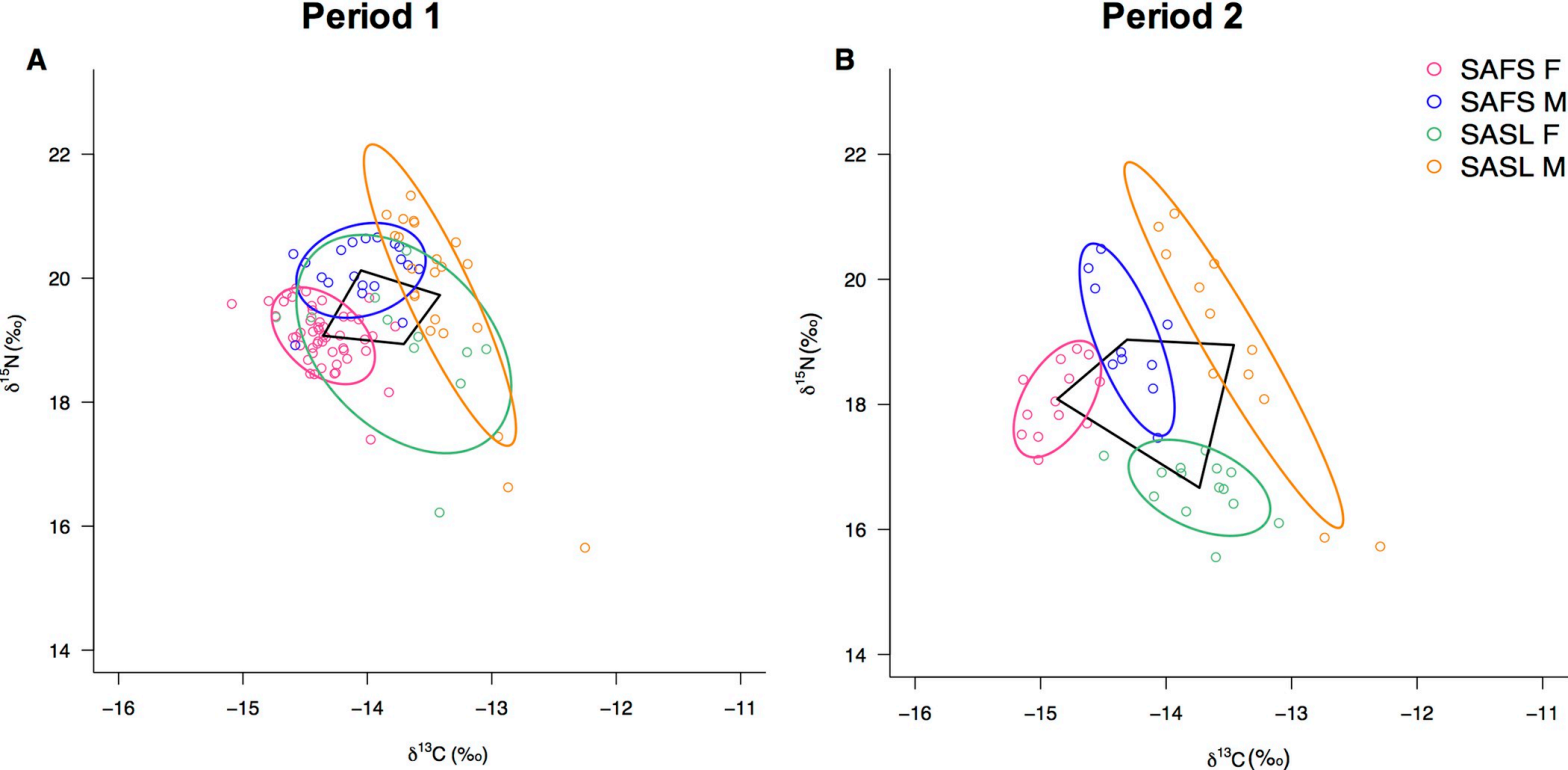
Standard Ellipse Area (75%) for A) Period 1 and B) Period 2. Black lines delineate community convex hull between centroids of each group.

Standard ellipse areas (SEAc) increased in male groups of both species between Period 1 and Period 2 (11.03% in SAFS M and 16.27% in SASL M). In females, SEAc remained stable in SAFS and decreased for SASL by 72.75% (Fig 2, Table 3). In Period 1, SASL female SEAc overlaps with all groups (66.37–92.99%) followed by SASL male SEAc that overlap with SASL females (30.90%) and SAFS males (22.63%) and SEAc of SAFS males and SAFS females that overlap in range of 12.85–24.58% and 10.85–15.48%, respectively. In Period 2 only two cases of overlap of ≤1% were detected (Table 4) and all ellipses segregate in isotopic space in Period 2. All Layman metrics increase in Period 2, including δ^15^N range, δ^13^C range, TA, CD, MNND and SDNND (Table 5).

**Table 3:** Corrected Standard Ellipse Area (SEAc) for each period and group estimated with 75% maximum likelihood.

Group	SEAc Period 1	SEAc Period 2
SAFS F	0.94	0.95
SAFS M	1.29	1.45
SASL F	4.88	1.33
SASL M	2.27	2.64

**Table 4:** Percent overlap between Peruvian otariid groups according to Period.

		SAFS F	SAFS M	SASL F	SASL M
**Period 1**	**SAFS F**	-	14.94	80.67	0
	**SAFS M**	10.85	-	92.99	22.63
	**SASL F**	15.48	24.58	-	30.90
	**SASL M**	0	12.85	66.37	-
**Period 2**	**SAFS F**	-	1.03	0	0
	**SAFS M**	0.67	-	0	0
	**SASL F**	0	0	-	0
	**SASL M**	0	0	0	-

Values indicate the percent overlap for the group in the row.

**Table 5:** Layman metrics estimated according to period.

Layman Metrics	Period 1	Period 2
δ^13^C range	0.94	1.41
δ^15^N range	1.18	2.36
TA	0.67	1.91
CD	0.60	1.05
MNND	0.71	1.16
SDNND	0.05	0.45

δ^13^C range = distance range of δ^13^C values, δ^15^N range = distance range of δ^15^N values, TA = Total area of convex hull, CD = the mean distance to centroid from means, MNND = mean nearest neighbor distance of the means, SDNND = standard deviation of the nearest neighbor distance.

## Discussion

In this study, changes in the niche of different species and sexes through time were investigated by examining the stable isotope ratios serially archived in whiskers of male and female South American fur seals and South American sea lions during 2005–2019 in the Humboldt Current System. We found a reorganization of our four otariid study groups in the isotopic space as a response to the shift towards warmer oceanographic conditions from November 2013 onwards, as depicted by the increase in SLA and SST (Period 2, Fig 1). Groups expanded their niche width (except SASL females), they segregated in isotopic space, and the δ^15^N signal was of lower value during the warmer period. Overall decline in otariid δ^15^N suggests impoverished baseline values of δ^15^N with bottom-up effects impacting tertiary consumers. Overlap reduced between all otariid groups in Period 2, providing support of a temporal shift from a more redundant assemblage (similar isotopic niches) during the colder period to more diversified foraging tactics with divergent isotopic niches during the warmer period.

Although we did find significant differences to compare the four species-sex groups according to morphometrics, mass was not a driver for higher δ^15^N as hypothesized, supporting findings in other studies where body mass is a poor predictor for trophic position in marine mammals [61,62]. In our study, differences in the isotopic signatures in both species might be driven by environmental conditions and behavioral traits according to sex. Different behavioral constraints between sexes in both otariid species may explain how larger male individuals invest more time and energy foraging at sea, possibly being more selective hunters to capture prey of higher trophic level, as evidenced in reports of diverse hunting strategies in male and subadult male Galapagos sea lions [63,64]. Although previous work in a captive male sea lion has found cyclical drops related to their fasting periods [65], the male isotopic changes here reported are not cyclical and have a higher magnitude. Notwithstanding, we do acknowledge that a source of variation could exist in whisker growth rates according to species [66]. However, the estimate applied to this study is within the reported range and variation of the applied value would not significantly alter the results.

Interestingly, sexual differences are generally maintained, but all δ^15^N values decrease in Period 2, with female SASL showing the largest drop in δ^15^N of 2.4‰, while male group’s values drop in 1.5‰ and female SAFS decrease in 1.0‰. We consider that the change in δ^15^N in Period 2 reflects a bottom-up effect in the trophic food web, linked to the dietary habits of each species and sex group, in response to changes in the environment. In November and December 2013 elevation in the SLA and SST was detected respectively, marking a regime shift towards warmer conditions in the sea surface and a deeper thermocline. This change is in accordance with the arrival of a downwelling Kelvin wave to the Peruvian coast and the onset of regional warming south of Peru, as reported by the Peruvian National Commission for the Study of El Niño Phenomenon [67] Afterwards, in mid-2014 a short moderate El Niño event was detected for the El Niño 1+2 Region [68] and for the Peruvian coast, which was followed by the large 2015–2016 El Niño [69]. Downwelling Kelvin waves that hit the South American coast then propagate poleward as coastal trapped waves, deepening the thermocline and thus reducing the upwelling of nutrient- rich waters, which in turn result in a decrease in productivity in the euphotic zone [45]. In the California Current, another upwelling eastern boundary current marine ecosystem, deeper chlorophyll-a decreased phytoplankton biomass and caused a shift in community structure towards a smaller phytoplankton species, which caused bottom-up effects on food and energy availability at higher trophic levels, that resulted in high mortality rates of red tuna crabs (*Pleuroncodes planipes*) and California sea lions (*Zalophus calfornianus*) [32]. Based on the long-term changes of the δ^15^N signal captured in otariid whiskers, we suggest that a similar bottom-up effect may have occurred in the Humboldt Current System off the coast of Peru.

The red squat lobster (*Pleuroncodes monodon*) inhabits the northern Humboldt Current System and has been abundant since the mid-1990s [70]. It has been identified to have both a pelagic and benthic lifestyle [71] and is an important prey item for seabirds, mammals, and coastal fish [72,73]. This crustacean has been identified as the dominant prey item in SASL diet composition (~70%) based on scat analysis, and the remainder are anchovies (19%) and squids (5%) [74]. In contrast, SAFS have been reported to consume mostly Peruvian anchovy (43%), squids (35%), myctophids (7%) and red squat lobsters contribute only a minor amount (5%) to the diet [14,15,74–76]. In the literature, there is conflicting information about the trophic levels of anchovies and red squat lobsters compared. In Espinoza et al (2017), sampling data from 2008–2011 (Period 1) reveals similar δ^15^N values for anchovies and red squat lobsters (13.7‰ and 13.6‰, respectively), proving difficult to interpret switch in prey items based on δ^15^N values. However in a recent study by Massing et al (2022) based on samples from 2018–2019 (Period 2), anchovies δ^15^N had a range of 13.8–22.8‰, making it at least 3‰ higher than the δ^15^N value of adult squat lobster (8.9‰). Also, prey items that are feeding closer to the oxygen minimum zones (OMZ) have been found to have higher δ^15^N values than those at the surface [71]. Therefore, variations in sampling seasons, latitude, depth and isotope turnover rate of different tissues could be influencing these different results. Nitrogen cycling in OMZ can have drastic and dynamic effects on the nitrogen left in marine upwelling ecosystems. During warming events or marine heat waves, intensified stratification and oxygen depletion can exacerbate nitrogen loss in Peru´s coastal upwelling system [77]. There is evidence that during El Niño events, diatoms, the dominant phytoplankton in the Humboldt Current System are greatly impacted by the less nutrient-enriched conditions, with a reported decrease of 25–60% of their biomass during these events, while the predominantly mixotrophic dinoflagellates can temporarily occupy their niche [78]. Meanwhile, zooplankton is the main prey item of anchovies [79] and enforces bottom-up control [80]. Thus, since SASL are feeding directly on red squat lobsters aggregated along the cold coastal waters, their δ^15^N signals could be expected to be lower in comparison to SAFS feeding on anchovies and squid in the pelagic realm [74].

In this ecosystem, regional baseline shifts have been found along the latitudinal range with higher δ^15^N values south of latitude 14°S in the water column [45,71]. In terms of the spatial extension, sampled otariids are expected to have a home range operating between 14°S -18°S at most, so this should not be an important source of variability. However, coupling benthic and pelagic habitats by prey items such as red squat lobsters and zooplankton species (i.e., copepods and krill) occurs by transferring carbon from anoxic seafloor conditions to the oxygenated surface layer, and can also be playing an important role in the isotope ratio signal [71]. Thus, complex nitrogen cycles and high variability in Humboldt Current System complicate interpretation of δ^15^N signal over large spatial and temporal scales.

The δ^13^C isotopic signature serves as a proxy for the inshore /offshore location of the foraging grounds. We found interspecific differences in δ^13^C with lower δ^13^C values in SAFS compared to SASL, independent of period. We did not find differences in δ^13^C were mainly explained by sex, but rather followed the inshore-offshore gradient reported by Espinoza et al. (2017), with decreasing trend in δ^13^C with increasing sampling distances from shore. We found lower δ^13^C values in SAFS that we consider are linked to more offshore resource acquisition in comparison to SASL. Furthermore, in the warming period, SAFS exhibit decreases in the δ^13^C signal towards offshore waters, while SASL remains in the same range. This agrees with the association of the biomass of the main prey item of SASL the red squat lobster with cold coastal waters that contract further inshore during warming events [81]. Studies using hydro acoustic surveys along the coast of Peru found that distribution of Peruvian anchovy and red squat lobsters spatially overlap, with both prey species distributed close to the coast, but red squat lobsters concentrate closer towards shore, in the first 40 km [70]. In contrast, Peruvian anchovies, the main prey item for SAFS, show a larger range in δ^13^C values, indicative of a wider use of the pelagic habitat in comparison to red squat lobsters [45]. Contrary to what we expected, between sexes of the same species, males did show slightly higher (less negative) δ^13^C values than females, which may reflect inclusion of coastal/benthic foraging in males. The latter can be related to the requirement in females for homogenous prey patches that are available in the offshore pelagic environment in the Humboldt Current System to more efficiently optimize milk production to nurse pups, whereas males have more possibilities of exploring different foraging grounds and strategies [82].

The reduction in overlap of the isotopic niches between the two periods suggests that the response to higher SLA (e.g., deeper thermocline) and warmer SST promoted trophic segregation between otariid predator communities, contrary to what we hypothesized. Layman metrics show that the ranges of both isotopic signatures (δ^15^N and δ^13^C ranges) and the total area occupied (TA) expanded in response to warmer conditions in Period 2 (S1 Fig). Furthermore, the lower levels of the trophic position in the otariid community during Period 2 was predominantly driven by a niche shift in all groups, with a significant contribution from SASL females (S2 Fig). This supports the theory that basal resources more depleted in 15N may have been predominant in Period 2, as reported in other studies showing temporal niche shifts in marine predators [2,83].

In general, populations can be separated into specialists when they have narrower niches or into generalists when they have broader niches [84]. Ecological specialists with evolve in relatively homogenous environments (in space and time), meanwhile generalists evolve in heterogenous environments [85]. Thus, during the warmer period, it is possible that prey assemblages are more diverse in contrast with dense prey aggregations favored by nutrient rich, cold-water in the Humboldt Current System. Since TA serves as a proxy for the extent of trophic diversity, its expansion (along with CD, NND and SDNND) reflects a diversification in the resources used by a community of more generalist species-sex groups, with increased trophic segregation between them during Period 2 [4]. Of all the groups, female SASL show the largest variation in response to the warming scenario, reducing both ellipse area and δ^15^N values. This suggests that this group has higher plasticity to adapt to changing environmental conditions, as reported in other otariids [83,86]. This helps explain current, larger population biomass of SASL reported for PSJ and Peru, in contrast to the smaller and steeper declining SAFS population [87]. Thus, plasticity and feeding on a more available food source that lacks commercial fishing pressure can be currently more advantageous for SASL.

In the Atlantic, different niche widths have been reported for SAFS and SASL according to location. In Isla Lobos Uruguay, SAFS have wider niches compared to SASL and are composed by generalist and specialist individuals [5,40]. Whereas in Falkland Islands, SAFS are considered specialists with a narrow niche and SASL generalists based on the combination of specialized inshore and an offshore groups [7]. Therefore, in these central place foragers, the environment is a key driver for the use and partition of local marine resources; which will vary according to location. However, in all the cases in the Atlantic there is minimal or no overlap between SAFS and SASL niches; even in studies including fossil records from the Holocene to the present [88]. This contrasts with our study, which evidences overlap during Period 1 which suggests that trophic overlap is possible between species and sex groups in more productive environment (Period 1), and that this can then change according to dynamic environmental conditions (Period 2).

Atlantic Ocean marine ecosystem is relatively stable compared to the dynamic and variable upwelling the Humboldt Current System. In the Galapagos Islands, an upwelling system with more similarities to the Humboldt Current System environment, segregation with low overlap exists between sympatric fur seals and sea lions. Galapagos fur seals (*Arctocephalus galapagoensis*) forage in the pelagic zone with a smaller population isotopic niche compared to benthic foraging Galapagos sea lions (*Zalophus wollebaeki*) with larger isotopic niche [89]. In our study site in the Peruvian Humboldt Current System, the bathymetry deepens very rapidly, reaching over 1000 m in less than 50 km offshore and the reduction in sublittoral benthic biomass and diversity due to oxygen deficiency, explains reduced benthic foraging. Therefore, the contribution of specialized individuals employing benthic strategies is still put in question. However, individual specialization and the capacity to adapt different foraging strategies can also be playing an important role in the variability between our study groups. For example, consumption of prey items, such as red squat lobsters that feed either at the surface or in the benthic realm within the OMZ can reflect different δ^15^N and δ^13^C values [71]. Therefore, we recommend future research explore individual specialization to understand exploitation mechanisms by otariids in the Humboldt Current System and how individuals respond to changing environmental conditions, taking into account the dynamic oceanographic conditions with complex nitrogen cycling.

## Supporting information

S1 Fig Standard ellipse area estimated for each group. Labels represent Community (Period 1 and 2) and Group (1 = SAFS females, 2 = SAFS males, 3 = SASL females, 4 = SASL males). The black points correspond to the mean standard ellipse area for each group, red cross is the standard ellipse area corrected for small sample size. Grey and white boxed areas reflect the 95, 75 and 50% confidence intervals.
(DOCX)

S2 Fig Total area of convex hull estimated for each community or period. Community 1 = Period 1, Community 2 = Period 2. The black points correspond to the mean standard ellipse area for each group while the grey and white boxed areas reflect the 95, 75 and 50% confidence intervals.
(DOCX)

S1 Table Change point analysis statistics. Location of point change detection in the time series, mean value for each period, test statistic and MBIC penalty value for each environmental condition.
(DOCX)
