# Supplementary material for: Sympatric otariids increase trophic segregation in response to warming ocean conditions in Peruvian Humboldt Current System
Source: PLoS One. 2022 Aug 11;17(8):e0272348. doi: 10.1371/journal.pone.0272348 (PMC9371314; doi:10.1371/journal.pone.0272348)
Supplement: S1 Fig — Labels represent Community (Period 1 and 2) and Group (1 = SAFS females, 2 = SAFS males, 3 = SASL females, 4 = SASL males). The black points correspond to the mean standard ellipse area for each group, red cross is the standard ellipse area corrected for small sample size. Grey and white boxed areas reflect the 95, 75 and 50% confidence intervals. (DOCX) [file pone.0272348.s001.docx]

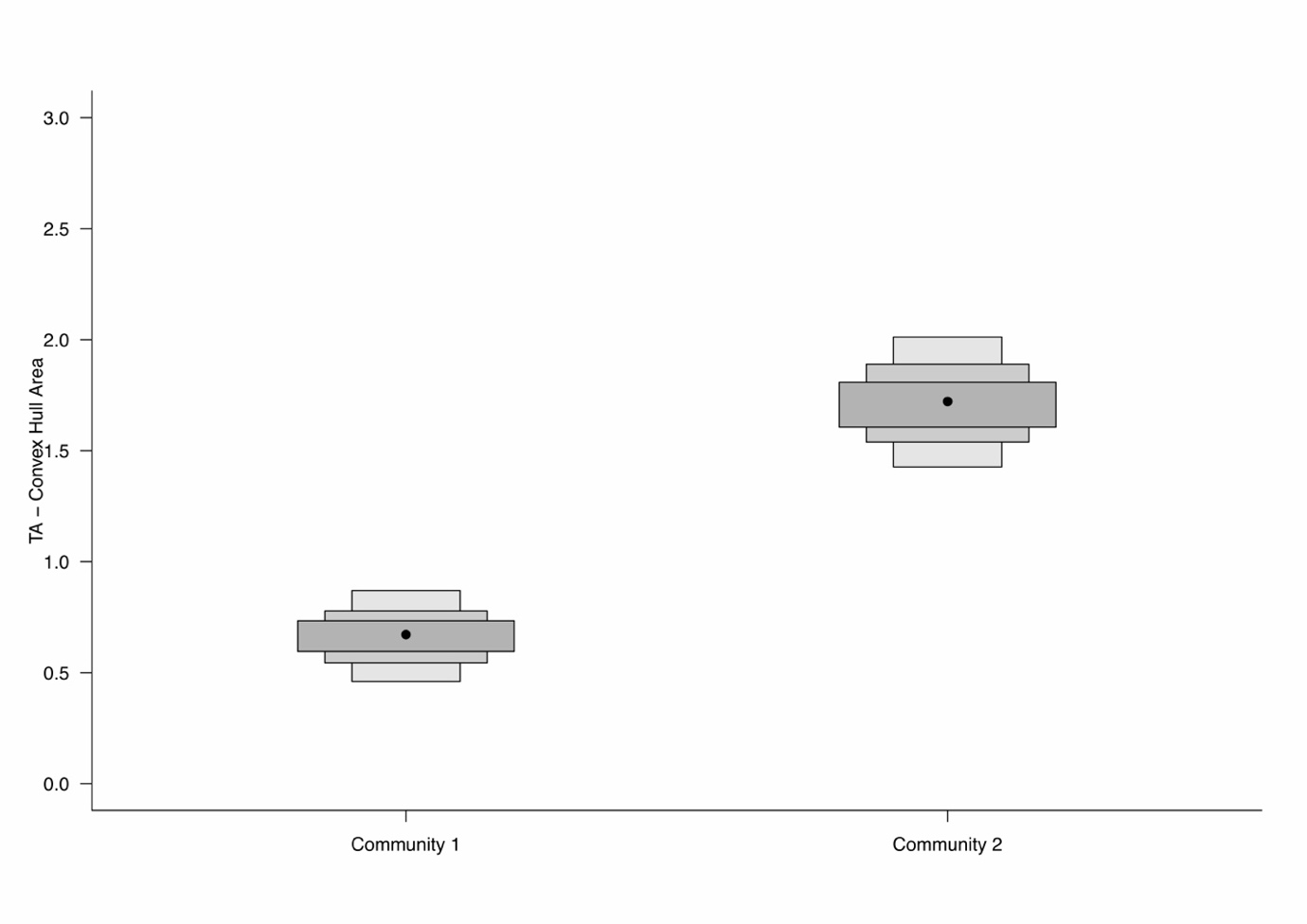


**S1 Fig.** **Total area of convex hull estimated for each community or period.** Community 1 = Period 1, Community 2 = Period 2. The black points correspond to the mean standard ellipse area for each group while the grey and white boxed areas reflect the 95, 75 and 50% confidence intervals.
