# Supplementary material for: Sympatric otariids increase trophic segregation in response to warming ocean conditions in Peruvian Humboldt Current System
Source: PLoS One. 2022 Aug 11;17(8):e0272348. doi: 10.1371/journal.pone.0272348 (PMC9371314; doi:10.1371/journal.pone.0272348)
Supplement: S1 Table — Location of point change detection in the time series, mean value for each period, test statistic and MBIC penalty value for each environmental condition. (DOCX) [file pone.0272348.s003.docx]

**Supplementary Information**

**S1 Table. Change point analysis statistics.**  Location of point change detection in the time series, mean value for each period, test statistic and MBIC penalty value for each environmental condition.

| **Parameters** | **Change point location** | **Period 1- Mean** | **Period 2- Mean** | **Test Statistic** | **MBIC Penalty value** |
| --- | --- | --- | --- | --- | --- |
| Sea Level Anomaly (SLA) | 107 (Nov 2013) | 3.42 | 8.06 | Normal | 15.57 |
| Sea Surface Temperature (SST) | 108 (Dec 2013) | 14.11 | 15.05 | Normal | 15.57 |
